# Burnout, recovery, and flow in youth high-performance athletes: a multilevel mixed-methods examination of mental health and psychological functioning in Chinese athletes

**DOI:** 10.3389/fpsyg.2026.1835056

**Published:** 2026-07-22

**Authors:** YiNan Zhang, JingHao Zhang, XueJun Li

**Affiliations:** 1 College of Innovation Management, Suan Sunandha Rajabhat University, Bangkok, Thailand; 2 Department of Physical Education, Shandong Technology and Business University, Yantai, Shandong, China; 3 Department of Physical Education Teaching and Research, Central South University, Changsha, Hunan, China

**Keywords:** athlete burnout, flow state, multilevel analysis, recovery experience, youth athletes

## Abstract

**Introduction:**

Youth athletes with extremely high accomplishments are under intense training that predisposes them to experiencing stress and emotional problems. This study sought to explore the relationship between burnout, recovery, flow, and psychological functioning among youth high-performance athletes in Guangdong province, China, employing a sequential explanatory mixed-methods approach.

**Methods:**

Quantitative data were collected from 450 youth high-performance athletes, while qualitative data were collected from 35 youth athletes and 12 coaches using reflexive thematic analysis. Quantitative analysis involved correlation, structural equation modeling, and multilevel regression.

**Results:**

Training load (β = 0.342, *p* < 0.001) and perceived stress (β = 0.298, *p* < 0.001) positively predicted athlete burnout. Recovery experience (β = −0.324, *p* < 0.001), flow experience (β = −0.276, *p* < 0.001), coaching support (β = −0.281, *p* < 0.001), resilience (β = −0.196, *p* < 0.001), and sleep quality (β = −0.173, *p* < 0.001) had a significant negative relationship with burnout. Fit indices indicated satisfactory construct validity (CFI = 0.947, TLI = 0.939, RMSEA = 0.051, SRMR = 0.047). Mediation analysis showed significant indirect effects of recovery on the training load-burnout relationship [β = 0.121, 95% CI (0.067, 0.183)], and of flow on the coaching support–burnout relationship [β = −0.109, 95% CI (−0.158, −0.061)].

**Discussion:**

Four themes emerged from the qualitative data — training stress, recovery control, flow, and coaching environment — reinforcing the quantitative findings that psychological recovery and optimal-performance states are key protective mechanisms against burnout in youth high-performance sport.

## 1 Introduction

Youth sport is an increasingly popular area in modern athletic development systems, especially in countries that invest much in the training of elite athletes at a young age (McLellan et al., 2022). Young athletes are subjected to intense training programs, strict competition requirements, and expectations to perform well in most competitive sport settings (Jayanthi et al., 2022; Bompa and Sarandan, 2022). Although the stated environments are meant to promote elite performance and international competitiveness, they also generate psychological stress that has a dramatic impact on the mental health and wellbeing of athletes. In this framework, the problem of chronic stress, emotional burnout, and the lack of motivation has become a burning topic in the youth sports community (Walton et al., 2024; Gwyther et al., 2024). The given experiences are traditionally interpreted via the prism of Athlete Burnout as a psychological state, which is defined by emotional and physical fatigue, the loss of athletic achievement, and the development of the devaluation of sport (Madigan et al., 2022; Marangoni et al., 2023). The etiology of burnout and ways in which it has been averted have thus taken a significant interest in the study of sport psychology.

The developmental issues of youth high-performance athletes are highly individualized since they have to manage the requirements of sport training at the elite level with the roles of educational, social, and personal development (Jiang et al., 2024; Holden et al., 2025). At this stage of adolescence and early adulthood, human beings experience tremendous psychological and emotional shifts, which render them highly vulnerable to the effects of environmental stressors (Sisk and Gee, 2022). In case athletes train within very competitive systems and focus on their results, the stress of expectation has also caused extended psychological stress. It has been found out that insufficient training loads, absence of recovery, and strict coaching organization has make young athletes more susceptible to burnout (Guard, 2025; Huang et al., 2025). As a result, researchers and practitioners have started to highlight the significance of recovery measures and psychological resources that has enable athletes to be well and at the same time ensure a high level of performance.

Psychological and physiological recovery, in turn, is one of the most significant protective mechanisms against burnout (Khammissa et al., 2022). Provided that recovery possibilities are lacking, over time, athletes has experience more stress, making them more susceptible to exhaustion and issues with psychological wellness (Rogers et al., 2024). Over the recent years, sport psychologists have come to the realization of the significance of recovery experiences that incorporate relaxation, mental disconnection with sport-related demands, and participation in restful activities (Rowlands et al., 2025). The processes assist the athletes in managing stress and ensuring that there is long-term psychological balance in the demanding systems of sport that are demanding.

Along with recovery processes, the other significant psychological construct that is relevant to positive athletic functioning is the experience of optimal engagement during sport participation (Raimundi et al., 2024). Athletes who have flow state report that they perform better, feel more motivated, and are more psychologically satisfied with their sports involvement (Bayram et al., 2025; Sánchez Vara et al., 2023). The probability of flow, however, has been diminished whenever the athletes are affected by stress, exhaustion, or psychological stress, which underscores the need to have balanced training conditions that facilitate performance and wellbeing.

These questions are especially topical in the framework of the development system of elite sports in China, which has traditionally focused on the identification of talents early and on the intensive training routes. The Chinese young athletes are also accustomed to training in special sport academies and provincial training centers where the main goal is the competitive success on the national and international levels (Chen and Chen, 2022). Although this system has yielded incredible sporting success, there have been growing questions among scholars about the psychological burdens of playing in such systems among young players. Long working hours, high performance standards, and insufficient opportunity to rest and develop personally has led to the situation where the level of stress increases and influences the mental health conditions. Although international concerns over athlete wellbeing have increased, empirical studies on the psychological experiences of Chinese youth high-performance athletes are still fairly scarce, especially as far as the relationship between burnout, recovery, and flow is concerned.

This being the case, the current research aims at analyzing the psychological operations of youth high-performance athletes by evaluating the interactions between training demands, recovery mechanisms, and motivational experiences to ascertain how these elements affect exhaustion. The multilevel mixed-methods approach has helped the research to embrace both statistical trends and lived experiences in the context of elite sport settings. In particular, the research investigates the mediating role of recovery experiences and flow states in the association between training-related stressors and athlete burnout, as well as the much wider contextual factors, including coaching relationships and psychological resilience. By conducting this in-depth analysis, the study has helped to gain a better insight into the mechanisms that underlie the sustainable development of athletes and their mental health in high-performance youth sport systems.

## 2 Literature review

Burnout among athletes is a complex psychological phenomenon that manifests itself through feelings of emotional and physical exhaustion, decreased sense of achievement, and sport devaluation due to chronic pressure from training and competition (Raedeke, 1997; Raedeke and Smith, 2001). This concept is based on burnout theory (Maslach and Jackson, 1981) and the cognitive-affective model of athletic burnout (Smith, 1986). In this context, burnout is viewed as an adaptive process to prolonged stress experienced by athletes who view demands as overwhelming their coping abilities. Constructs related to burnout include concepts of resilience, recovery, coping, motivation, and flow. Specifically, resilience can be defined as an ability to successfully cope with stress situations (Fletcher and Sarkar, 2012), while recovery implies psychological and physiological restoration following stressful experiences (Kellmann, 2010). Coping is seen as a set of cognitive and behavioral processes aimed at regulating stress (Lazarus and Folkman, 1984), motivation is described as quality of behavioral regulation in sports (Deci and Ryan, 1985), and flow refers to the state of optimal psychological functioning associated with total immersion into the task and enjoyment of its performance (Csikszentmihalyi, 1990).

There is a large research base in sport psychology that points to a complex interaction between the factors of stress, coping, motivation, emotional regulation and contextual factors as contributing to the development of burnout in athletes (Gustafsson et al., 2011; Goodger et al., 2007). Early research confirmed that chronic stress, the feeling of stressor imbalance, and poor coping strategies are significant factors in athletes’ burnout (Smith, 1986; Raedeke and Smith, 2001). Emotional regulation has since become a protective factor, with athletes who have a higher capacity to be emotionally clear and emotionally repair being better able to manage the stress of the competition, and to avoid the maladaptive response. In line with this view, Zuo and Bai (2025) reported that the negative correlation between emotional regulation capabilities and burnout was found in Chinese athletes, with coping partially explaining the link between self-blame and denial and the link between the two and burnout.

Cooperating theory has always been on the premise that psychological results not just rely on the stressor exposure but additionally upon how the individual appraises and deals with the stressors (Lazarus and Folkman, 1984). In sport, adaptive coping (problem-focused coping and cognitive reappraisal) has been associated with decreased exhaustion and increased functioning, while avoidance-oriented coping has been associated with maladjustment (Nicholls and Polman, 2007). This is supported by recent evidence. According to Mei et al. (2025), there was a significant relationship between resilience and the reduction of burnout, and the relationship was mediated by the coping strategies of problem-oriented and avoidance-oriented coping strategies, as well as Xu A. et al. (2024) and Xu H. et al. (2024) showed that coping strategies and intrinsic motivation were responsible for the relationship between perfectionistic concerns and exhaustion.

The study also determined that other factors that could buffer against athlete burnout are resilience and psychosocial support. Resilient athletes are better able to sustain their psychological functioning in challenging environments and have a more adaptive psychological reaction to a performance challenge (Fletcher and Sarkar, 2012). Oleas D. et al. (2025) and Oleas M et al. (2025) reported that social support was associated with a decrease in mental fatigue and burnout, especially for younger athletes, whereas mental hardiness was associated with a decrease in mental exhaustion. Likewise, positive psychological resources like self-compassion, confidence and mindfulness have been linked to enhanced wellbeing and lowered burnout danger (Wang N. et al., 2025; Wang Y. et al., 2025; Yiyi et al., 2025).

Other mental health signs also impact on burnout and performance. Athlete burnout has been found to have both direct and indirect links with perceived stress, anxiety and depression (Gao Y. and Wang L, 2024; Gao X. and Wang Y, 2024), and psychological wellbeing is found to mediate relationships between training conditions/recovery strategies, motivation, and athletic performance (Liu H. and Fu S, 2024; Liu P. and Fu Z, 2024). Another argument in favor of recovery theory is that inadequate recovery from training stress leads to psychological fatigue and hence to a decrease in performance (Kellmann, 2010).

Factors of environment and relation are also influential. Athlete psychological outcomes and performance trajectories are influenced by the quality of coaching, autonomy supportive environment, training intensity, and interpersonal relationships (Deci and Ryan, 1985; Yang P. et al., 2024). Coaching behaviors, however, that are abusive, in contrast, raise the likelihood of burnout, particularly when coach-athlete relationships are poor (Li et al., 2025a,b). Furthermore, the impact of flow experiences and state mindfulness on positive mood, vigor and self-rated performance in dynamic sport settings has been demonstrated (Csikszentmihalyi, 1990; Zhang et al., 2024).

Developmental considerations also suggest that the transition to adolescence is a period when athletes face specific stressors, including academic performance, identity and self-concept, and interpersonal relationships, which increases the importance of considering psychological adaptation in the context of environmental support and self-regulatory resources over the course of the adolescent years and life-long mental health pathways (Guo et al., 2025).

Previous studies have yielded valuable insights, but most of these studies have been conducted on a one-level basis or on an individual basis (Quan et al., 2025; Zhang et al., 2025). Very few studies, however, have tried to address training environments, recovery, experiences of flow, and burnout outcomes within an overall framework of athletic performance in high performance youth athletes. Given this, the present study aims at filling this void by utilizing a multi-level mixed methods approach investigating the interrelated psychological and contextual processes that lead to burnout, recovery and flow in high-performance young Chinese athletes (Chen et al., 2024; Wu et al., 2014).

### 2.1 Theoretical framework and hypothesis development

In the present study, three complementary theories—Self-Determination Theory (SDT), Flow Theory, and Conservation of Resources (COR) Theory—are combined to understand the psychological processes and mechanisms that contribute to athlete burnout in high-performance youth sport settings. All of these theories independently account for important aspects of an athlete’s functioning, but together can offer a more complete multilevel explanation of how environmental demands, motivational processes, and psychological resource dynamics all contribute to the development of burnout. SDT (Deci and Ryan, 1985; Ryan and Deci, 2000) focuses on how social environments affect motivation and wellbeing by meeting or thwarting the basic psychological needs for autonomy, competence and relatedness. Optimal performance states can be described by the Flow Theory (Csikszentmihalyi, 1990; Csikszentmihalyi et al., 2014) as deep involvement into the task, intrinsic enjoyment, and absorption. According to COR Theory (Hobfoll, 1989; Hobfoll et al., 2018), stress and burnout are the result of lost resources, inadequate recovery and a lack of replenishment of psychological and physical resources. Together, SDT, Flow Theory and COR Theory account for why athletes get motivated or demotivated, why optimal experience helps to protect engagement in performance, and why resource depletion can result in exhaustion and burnout (Ryan and La Guardia, 2000; Wendling et al., 2018). They make up a sequential logic: environmental demands influence motivation (SDT), which in turn affects the engagement quality (Flow), and repeated exposures to demands without recovery cause resource loss spirals (COR) which can eventually result in burnout (Sarkar and Fletcher, 2013; Stamatelopoulou et al., 2018).

In SDT, athlete burnout is viewed as a result of the chronic need frustration that can happen in high-demand or controlling sport situations (Ryan and Deci, 2017). Elite youth sport systems can negate the sense of autonomy and competence by fostering training environments with high workload and externally controlled demands, which may lead to increased risk of feeling exhausted and devaluated (Gagné and Deci, 2005). Training load is based on the summation of all physical and psychological demands that are placed through structured training sessions and has been consistently related to fatigue, emotional exhaustion and performance strain (Borresen and Lambert, 2009; Gabbett, 2016). However, an increased training load and insufficient psychological recovery can lead to maladaptive stress reactions and burnout symptoms. Based on SDT need thwarting mechanism and empirical research on overtraining, the following hypotheses were proposed:

*H1:* Training load positively affects athlete burnout in youth high-performance athletes.

In contrast, SDT posits that coaching environments which are autonomy supportive will foster intrinsic motivation and psychological wellbeing, meeting basic psychological needs (Ryan and Deci, 2000). Past empirical sport psychology studies have consistently found links between perceived coaching support and increased motivation, decreased stress and decreased burnout (Mageau and Vallerand, 2003; DeFreese and Smith, 2013a, Li et al., 2025b). Coaching support which is perceived by the athletes as emotional, informational, and instrumental support, fosters relatedness and competence satisfaction, consequently decreases the risk of burnout. Relationships between coaches and athletes are also found to have a buffering effect on the negative impact of competitive stress (Jowett and Cockerill, 2003). Thus, the hypothesis of this research is:

*H2:* Coaching support is negatively related to athlete burnout.

Resilience in the context of sport is mainly based on COR theory and sport resilience literature. According to COR Theory, people seek to attain, retain and defend those things that are valuable to them, and stress is an experience when the resources that are sought are threatened or lost (Hobfoll, 1989). The word resilience in sport psychology is defined as maintaining or restoring psychological performance under stress (Fletcher and Sarkar, 2012). Resilient athletes can better maintain their motivation and control under high demand—they can avoid spirals of resources that can cause burnout. Empirical results indicate that the relationship between resilience and exhaustion and performance fatigue is negative (Gustafsson et al., 2017). Therefore, the relationship between psychological resilience and athlete burnout is a negative one, as in

*H3:* Psychological resilience negatively predicts athlete burnout.

Within COR Theory, sleep quality is considered as a basic resource of recovery—both physiologically and psychologically. Sleep is critical for cognitive restoration, emotional regulation and physiological regeneration, all of which are important for maintaining athletic performance (Fullagar et al., 2015; Asplund C. and Chang C. J., 2020; Asplund C. A. and Chang L., 2020). The quality of sleep is important, as it affects attention, stress perception and recovery ability, and can increase the rate of resource depletion. Empirical research in elite athletes has shown a relationship between sleep disturbance and heightened feelings of fatigue, mood disturbance and greater risk for burnout (Walsh et al., 2021). Thus, athlete burnout negatively predicted by sleep quality is theoretically supported by the mechanisms of recovery depletion from the COR.

Perceived stress is viewed as a psychological response that occurs when demands in the environment are appraised (Lazarus and Folkman, 1984). At the sporting level, high perceived stress is an imbalance between demands and coping resources, leading to an increased risk of emotional exhaustion and burnout (Nicholls et al., 2016). In COR Theory, stress is an antecedent and amplifier of the processes of resource loss. Therefore,

*H5:* Perceived stress is a positive predictor of athlete burn out.

Central recovery mechanisms in COR Theory and the Recovery-Stress Balance Model (Kellmann, 2010) are recovery experience. Recovery is psychological detachment, relaxation, mastery experiences, and control periods outside of training that replenish depleted resources and minimize cumulative fatigue (Sonnentag and Fritz, 2007). Poor recovery creates a vicious cycle of resource loss and burnout, and good recovery breaks that cycle (Doherty et al., 2021). Therefore, it can be concluded that the recovery experience has a negative relationship with athlete burnout (H6).

The concept of flow experience is based on Flow Theory which describes optimal psychological functioning in the process of engagement with the task (Csikszentmihalyi, 1990). The three factors of flow—absorption, enjoyment, and intrinsic motivation—increase performance satisfaction and decrease cognitive strain. Empirical research indicates that flow states correlate with decreased anxiety, increased wellbeing, and decreased risk for burnout in athletes (Swann et al., 2017). Hence, the hypothesis that flow experience is negatively related to athlete burnout is accepted.

The mediation hypotheses combine SDT and COR Theory in a sequential motivational–resource approach. In addition to stress, training load also decreases recovery opportunities, resulting in resource depletion. The mechanism of insufficient recovery to restore energy resources is the notion of loss spirals as explained by COR Theory (Hobfoll et al., 2018). The buffering mechanism that breaks cycles of resource depletion is therefore recovery experience, which is the mediator in the relationship between training load and burnout. Empirical sport research confirms that the quality of recovery is a predictor of the effects of training on exhaustion (Kellmann, 2010; Sonnentag and Fritz, 2007). Therefore,

*H8:* Recovery experience moderates the training load-athlete burnout relationship.

Mediation through flow theory primarily relies on the integration of SDT and Flow Theory. Autonomy supportive coaching climates positively predict intrinsic motivation and need satisfaction, thus increasing the probability of flow experiences in training and competitions (Ryan and Deci, 2000; Engeser and Rheinberg, 2008). Flow positively predicts positive affect and lower perceived exertion, reducing burnout risks. Motivational climate positively predicts flow experiences that in turn predict psychological wellbeing (Stavrou et al., 2015).

*H9:* Flow experience mediates the relationship between coaching support and athlete burnout.

**FIGURE 1:**
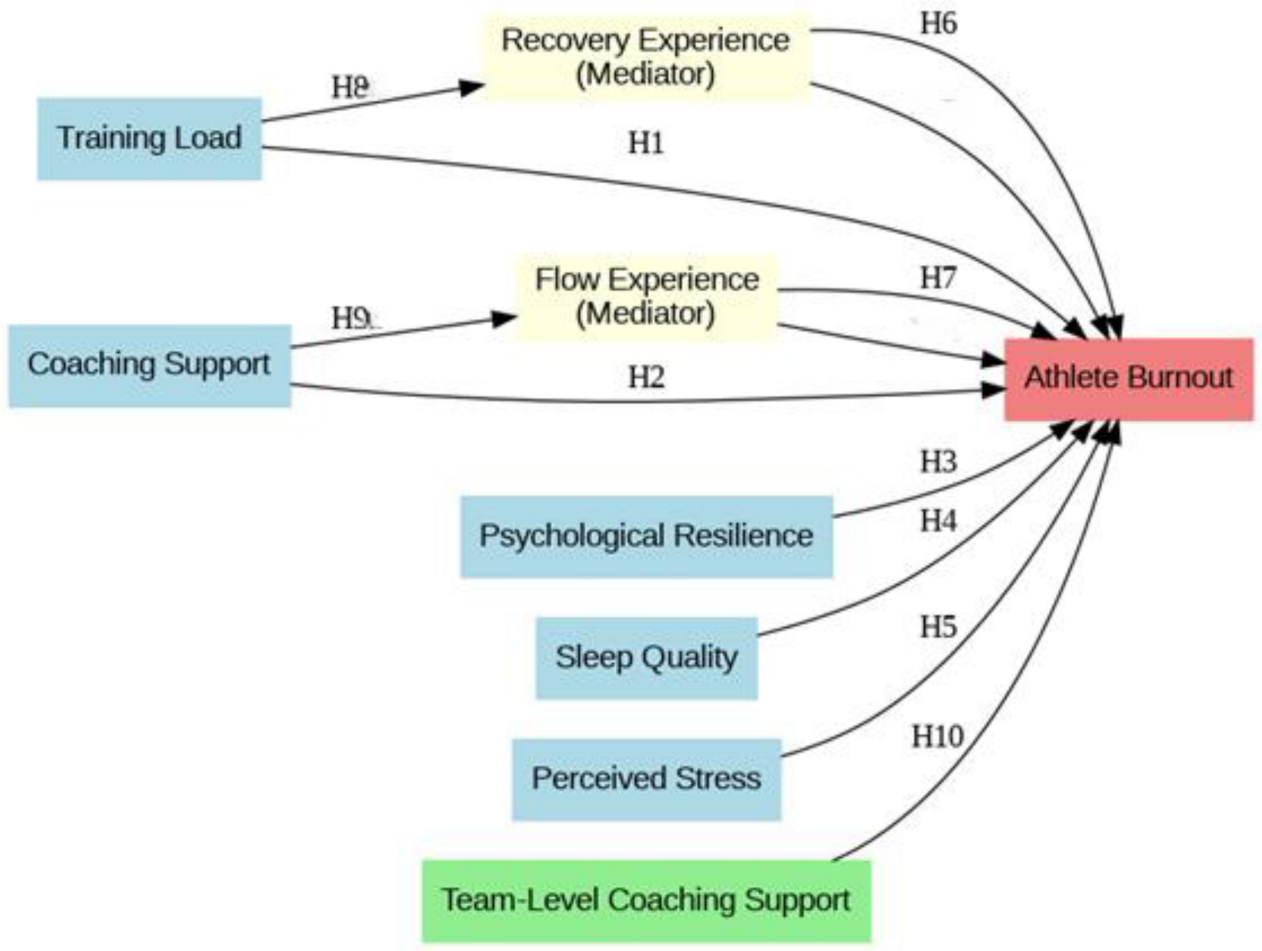
Conceptual framework diagram.

Lastly, the multilevel hypothesis is based on ecological models of sport psychology which recognize that athletes operate in teams. Coaching climate acts as a shared team-level resource for team-level psychological functioning. Higher team-level coaching support leads to higher shared motivation and lower burnout risk among athletes in the same setting (Jowett and Cockerill, 2003). Hence,

*H10:* Team-level coaching support negatively predicts athlete burnout at the group level. The hypothesized relationships among all study variables are summarized in the conceptual framework (Figure 1).

## 3 Research methodology

The study adopted the explanatory sequential mixed methods design to explore the interrelationships between burnout, recovery experiences, flow states, and psychological functioning in high-performance youth athletes in China. The design was based on two phases: Phase 1 was to collect and analyze quantitative data, and Phase 2 was to explain and elaborate on the quantitative data findings using qualitative data. This sequencing meant that qualitative inquiry was not generic, but rather was guided by the quantitative results and especially patterns of mediation between training stressors, psychological resources, and burnout outcomes that were unexpected. The merging and connecting of the two datasets took place in the interpretation stage, as a result of which a comprehensive understanding of psychological processes in contexts of elite sport was obtained.

The research population consisted of young athletes from the high-performance training system in Guangdong Province, China who were involved in the provincial or national training system aged between 14 and 21 years. The region was chosen because of its highly developed sporting facilities and the presence of high quality training centers. The quantitative sampling was adopted the multi-stage stratified random sampling method in three major institutions, including the Guangdong Provincial Sports Training Centre, the training system of Guangzhou Sport University and the Guangdong Youth Sports School. Athletes were divided into three groups, depending on sport type (endurance, skill based and team) to ensure the mix in training pressure and psychological stress. The a priori Monte Carlo power simulation for multilevel structural equation modeling resulted in a total sample size of 450 athletes, which provides power of ≥0.85 for detecting medium indirect effects (β = 0.20–0.30) with 5,000 bootstrap resamples at α = 0.05. This method succeeded in a more complex rule where each participant was allowed to contribute only a single parameter and provided a sufficient number of observations in nested mediation pathways.

During the qualitative phase purposive sampling was employed to identify 35 athletes and 12 coaches that varied maximally across sport type, competitive level, gender and training intensity exposure. Interviews were conducted until thematic saturation (no additional conceptual codes across three successive rounds of interviews) was reached at interview 41. Participant profiles were systematically recorded and included both individual (swimming, gymnastics, athletics) and team sports (basketball, volleyball, football) and were competitive at provincial and international junior level.

Burnout was defined as a multidimensional psychological syndrome characterized by feelings of emotional exhaustion, decreased sense of accomplishment and devaluation of the sport. It was assessed with a 4 item short form of the Athlete Burnout Questionnaire (ABQ), developed by Raedeke and Smith (2001). Through the review by experts and pilot testing, items were adapted to ensure that they are contextually relevant to Chinese youth athletes, yet conceptually faithful to the three core dimensions. The answers were given on a 5 point Likert scale, from strongly disagree (1) to strongly agree (5). Recovery experience was defined as the individual’s perception of psychological recovery after the training stress and was measured with a 4-item adapted version of the Recovery Experience Questionnaire (REQ) that assessed detachment, relaxation, mastery, and control. Flow experience was conceptualized as an optimal absorption and intrinsic engagement in sport tasks, and was assessed with the 4-item Dispositional Flow Scale-2 (DFS-2) measuring absorption, enjoyment, control and autotelic experience.

Training load was operationalized as the perceived physical and psychological training intensity with a 4 item version of the Session Rating of Perceived Exertion (sRPE) scale, which measures intensity, duration perception, fatigue response, and cumulative strain. Coaching support was assessed with a 4-item adapted Coach—Athlete Relationship scale (CART-Q) which focused on closeness, commitment, quality and perceived support. Psychological resilience was operationalized as the ability to continue functioning or recover from adverse circumstances, and was assessed through a 4-item Connor–Davidson Resilience Scale (CD-RISC), which focuses on adaptability, perseverance, emotional control and bouncing back from setbacks. The Perceived Stress Scale (PSS-10) was used to assess perceived stress with 4 items that cover unpredictability, overload, and lack of control. Sleep quality was assessed by a 4-item adapted Pittsburgh Sleep Quality Index (PSQI) which included the four following subscores: sleep latency, sleep duration, sleep disturbances, and subjective sleep quality.

All the instruments were translated and culturally adapted according to the WHO guidelines: forward translation, expert panel review, back-translation, and cognitive interviewing with athletes for semantic and conceptual equivalence. Minor wording changes were made, but no structural changes were made, after comparing a pilot study (*n* = 60) for clarity, reliability, and cultural appropriateness. The final sample was internally consistent (all constructs α > 0.78).

SPSS and AMOS were used for the analysis of quantitative data. Normality (skewness and kurtosis ≤ ± 2), linearity, and homoscedasticity assumptions were first tested before hypothesis testing. The presence of multicollinearity was determined through variance inflation factor (VIF < 3.0) and tolerance values (> 0.30). FIML was used to address missing data (< 3%) by assuming that data were missing completely at random (MCAR), which was confirmed through Little’s MCAR test. Confirmatory Factor Analysis (CFA) was conducted for measurement validity, with model fit determined using CFI (> 0.90), TLI (> 0.90), RMSEA (< 0.08), and SRMR (< 0.08).

A multiple regression model will be developed to evaluate the effect of training and psychological variables on athlete burnout.

Let: *Y* = Athlete Burnout (Dependent Variable); *X*_1_ = Training Load; *X*_2_ = Coaching Support; *X*_3_ = Psychological Resilience; *X*_4_ = Sleep Quality; *X*_5_ = Perceived Stress; *M*_1_ = Recovery Experience (Mediator); *M*_2_ = Flow Experience (Mediator)

The regression equation is:


$$Y=\beta_{0}+\beta_{1}\,X_{1}+\beta_{2}\,X_{2}+\beta_{3}\,X_{3}+\beta_{4}\,X_{4}+\beta_{5}\,X_{5}+\varepsilon$$

Where: β_0_ = Intercept; β_1_−β_5_ = Regression coefficients; ε = Random error term.

Mediation analysis tests whether recovery and flow explain the relationship between environmental factors and burnout.

### 3.1 Mediator model (recovery)


$$M_{1}=a_{0}+a_{1}\,X_{1}+a_{2}\,X_{2}+a_{3}\,X_{3}+a_{4}\,X_{4}+a_{5}\,X_{5}+e_{1}$$

Where: *M*_1_ = Recovery Experience; *a*_1_−*a*_5_ = Effects of predictors on recovery

### 3.2 Mediator model (flow)


$$M_{2}=b_{0}+b_{1}\,X_{1}+b_{2}\,X_{2}+b_{3}\,X_{3}+b_{4}\,X_{4}+b_{5}\,X_{5}+e_{2}$$

Where: *M*_2_ = Flow Experience

### 3.3 Outcome model (burnout)


$$Y=c_{0}+c_{1}\,X_{1}+c_{2}\,X_{2}+c_{3}\,X_{3}+c_{4}\,X_{4}+c_{5}\,X_{5}$$

$$+d_{1}\,M_{1}+d_{2}\,M_{2}+e_{3}$$

Where: *d*_1_ = Effect of Recovery on Burnout; *d*_2_ = Effect of Flow on Burnout

To assess the multilevel structure, a null model was employed to derive ICCs, revealing significant between-group variability on burnout (ICC = 0.21). Thus, hierarchical linear modeling was warranted. Maximum Likelihood (ML) with robust standard errors was applied for model estimation. The multilevel regression model is specified as follows:

Level-1 (Athlete level):


$$B\,u\,r\,n\,o\,u\,t_{i\,j}=\beta_{0\,j}+\beta_{1\,j}\,\left(T\,r\,a\,i\,n\,i\,n\,g\,L\,o\,a\,d_{i\,j}\right)+\beta_{2\,j}\,\left(R\,e\,s\,i\,l\,i\,e\,n\,c\,e_{i\,j}\right)$$

$$+\beta_{3\,j}\,\left(S\,l\,e\,e\,p\,Q\,u\,a\,l\,i\,t\,y_{i\,j}\right)+\beta_{4\,j}\,\left(S\,t\,r\,e\,s\,s_{i\,j}\right)+r_{i\,j}$$

Level-2 (Team level):


$$\beta_{0\,j}=\gamma_{00}+\gamma_{01}\,\left(C\,o\,a\,c\,h\,i\,n\,g\,S\,u\,p\,p\,o\,r\,t_{j}\right)+u_{0\,j}$$

Mediation effects were tested using bias-corrected and accelerated (BCa) bootstrapping with 5,000 resamples. The mediation model was correctly specified as:


$$B\,u\,r\,n\,o\,u\,t=c^{\prime }\,X+d_{1}\,\left(R\,e\,c\,o\,v\,e\,r\,y\right)+d_{2}\,\left(F\,l\,o\,w\right)+e$$

$$R\,e\,c\,o\,v\,e\,r\,y=a\,X+e_{1};F\,l\,o\,w=b\,X+e_{2}$$

where indirect effects were computed as *a*×*d*_1_ and *b*×*d*_2_, while *c*′represented the direct effect. This specification avoided conflation of total and direct effects.

Qualitative data collection was clearly informed by quantitative data, especially regarding mediation effects on recovery and flow. Semistructured interviews were based on four main categories: (1) training stress and fatigue perceptions, (2) emotional and cognitive coping mechanisms, (3) recovery methods and psychological detachment, and (4) experiences of being in an optimal state (flow). These interviews were carried out in Mandarin, lasted from 45 to 60 min, and were recorded with the participants’ permission. All interviews were transcribed verbatim and translated into English through a process of forward-backward translation.

Thematic analysis was done following the six-stage framework outlined by Braun and Clarke. Two independent coders performed thematic analysis of the transcribed interviews using the NVivo software. The initial open coding resulted in 84 codes that were subsequently clustered to yield 18 subthemes and 6 main themes. Intercoder reliability was calculated using Cohen’s kappa (κ = 0.82), which shows strong agreement. To enhance trustworthiness, member checks, peer debriefings, triangulation among athletes and coaches, and keeping an audit trail of the entire analysis process were done.

This process involved integration of the two approaches in the form of joint displays that compared the statistical paths with the thematic paths, giving qualitative data the ability to provide an explanation for the recovery and flow-mediated burnout relationships found in the SEM results.

## 4 Results

### 4.1 Demographic characteristics of respondents

The demographic profile of the respondents gives us a picture of how the high-performance athletes among the youth are composed during the study. There were 450 athletes in the analysis, as indicated in Table 1. The distribution of genders showed that there are 258 participants (57.3) who were males and 192 participants (42.7) who were females, which is relatively balanced with regard to the male and female athletes’ representation in the elite youth sports system. In terms of age distribution, the highest number of respondents was in the age group of 1,719 years (40.4%), then next was 1,416 years (32.4%), and lastly was the 2,021 years age group (27.2%). This distribution is representative of the age distribution of the high-performance youth sport development programs, with the majority of participants in these programs being young adults in late adolescence. Individual sports had the highest proportion of participants (45.6%), then team sports (35.6%), and skill-based sports (18.8), including racket and precision-based sports. This difference in the type of sports also offers a wide range of training conditions and performance needs. The experience of training also demonstrated that 38.7% of the athletes had between 3 and 5 years of training experience, 35.8% had between 6 and 8 years of training experience, and 25.5% had more than 8 years of training experience, which showed that the majority of the participants had a very long experience of working in competitive sport settings. Regarding the level of competition, almost half (49.6) of the respondents competed at provincial levels, 38.4% nationally and 12.0% internationally at the youth level, which is a relatively high level of athletic performance among the respondents.

**TABLE 1:** Demographic characteristics of youth high-performance athletes (*N* = 450).

Variable	Category	Frequency	Percentage (%)
Gender	Male	258	57.3
	Female	192	42.7
Age group	14–16 years	146	32.4
	17–19 years	182	40.4
	20–21 years	122	27.2
Sport type	Individual sports	205	45.6
	Team sports	160	35.6
	Skill-dominant sports	85	18.8
Training experience	3–5 years	174	38.7
	6–8 years	161	35.8
	Above 8 years	115	25.5
Competition level	Provincial	223	49.6
	National	173	38.4
	International youth	54	12.0

### 4.2 Descriptive statistics, reliability, and validity

**FIGURE 2:**
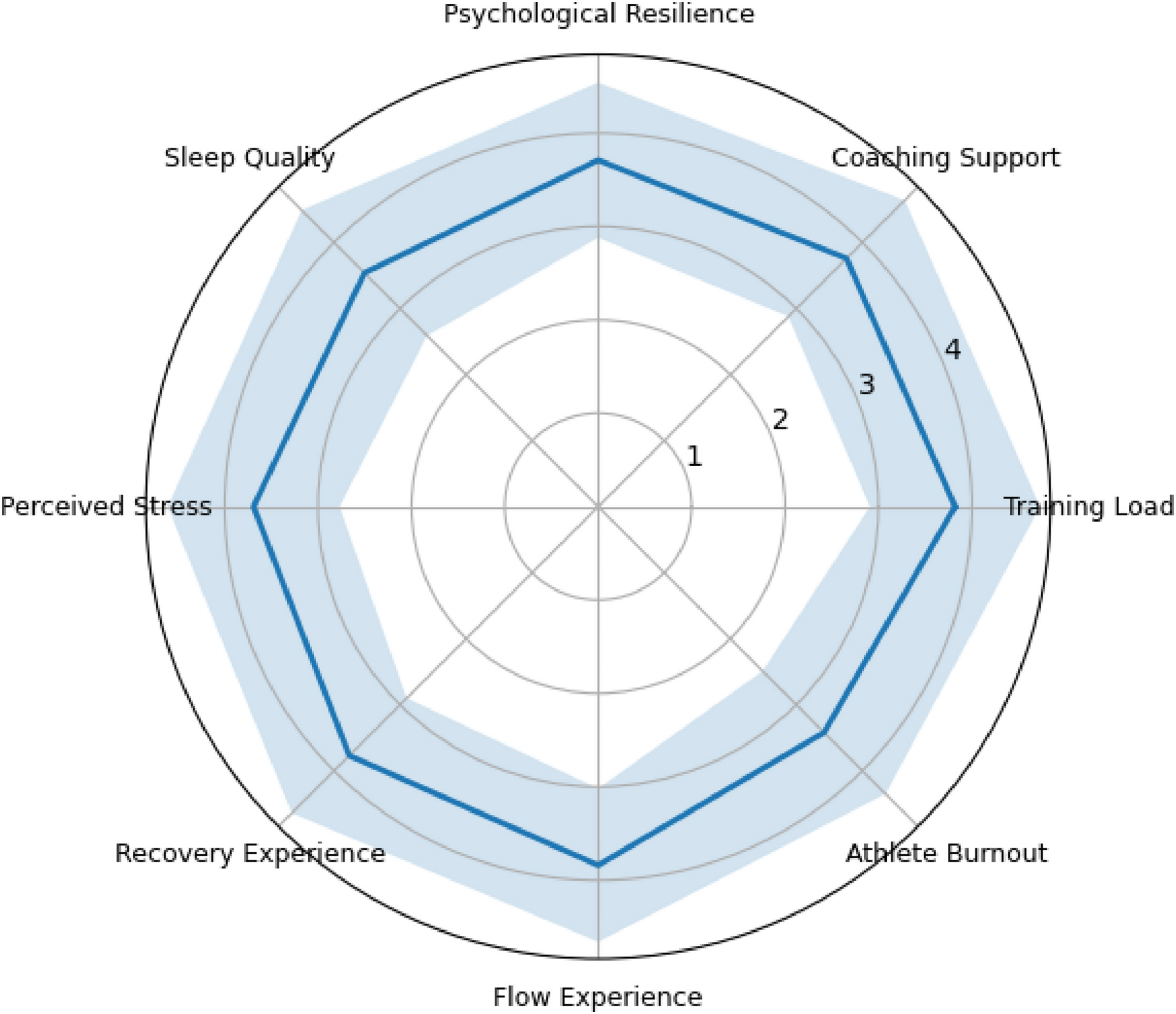
Mean scores.

To determine the distribution and internal consistency of the study variables, descriptive statistics and reliability analyses were done. The average of the variables is as shown in Table 2, with the mean between 3.42 and 3.84, which is moderate and high in most of the constructs. The highest mean score was found in flow experience (*M* = 3.84, SD = 0.82), training load (*M* = 3.82, SD = 0.91) and recovery experience (*M* = 3.77, SD = 0.87). On the other hand, the lowest mean score (*M* = 3.42, SD = 0.93) was recorded in athlete burnout, which implies that moderate levels of burnout are present in the sampled athletes. The reliability analysis revealed that there was a high level of internal consistency among the constructs. On the same note, composite reliability scores were between 0.885 and 0.928, meaning that there was excellent construct reliability. The convergent validity was also justified, which exceeds the required minimum of 0.50. These results prove that the measurement scales employed in the research have sufficient reliability and validity (Figure 2).

**TABLE 2:** Descriptive statistics and reliability.

Variable	Mean	SD	Skew	Kurtosis	Cronbach’s α	Composite reliability	AVE
Training load	3.82	0.91	−0.42	−0.37	0.872	0.891	0.673
Coaching support	3.76	0.88	−0.31	−0.28	0.886	0.902	0.698
Psychological resilience	3.71	0.83	−0.35	−0.22	0.894	0.909	0.714
Sleep quality	3.54	0.95	−0.28	−0.18	0.864	0.885	0.659
Perceived stress	3.69	0.92	0.41	0.36	0.879	0.896	0.681
Recovery experience	3.77	0.87	−0.39	−0.25	0.901	0.917	0.736
Flow experience	3.84	0.82	−0.44	−0.33	0.907	0.922	0.748
Athlete burnout	3.42	0.93	0.46	0.38	0.914	0.928	0.764

### 4.3 Measurement model assessment

**FIGURE 3:**
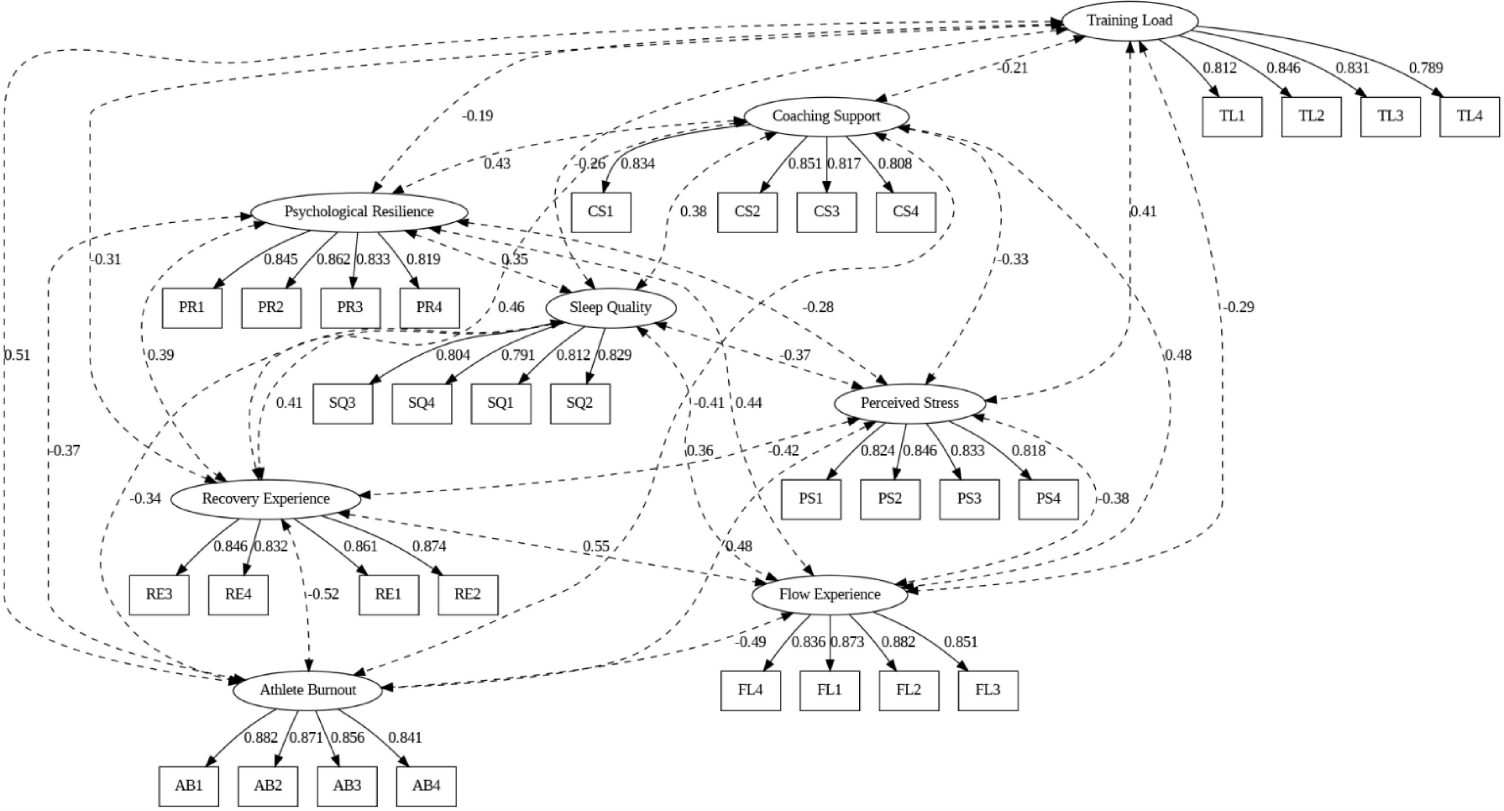
CFA measurement model.

Table 3 indicates that all the constructs had four observed indicators to measure them. Factor loading standardization was between 0.789 and 0.882, which is more than the recommended minimal value of 0.70. The flow experience item FL2 (0.882), then the athlete burnout item AB1 (0.882) and the recovery experience item RE2 (0.874) had the highest loading. These findings show the high indicator reliability and prove the convergent validity of the measurement model. In general, the measurement model exhibited a sound factor structure that has been used in the analysis of structural equation modeling (Figure 3).

**TABLE 3:** Measurement model with factor loadings.

Construct	Item	Factor loading
Training load	TL1	0.812
	TL2	0.846
	TL3	0.831
	TL4	0.789
Coaching support	CS1	0.834
	CS2	0.851
	CS3	0.817
	CS4	0.808
Psychological resilience	PR1	0.845
	PR2	0.862
	PR3	0.833
	PR4	0.819
Sleep quality	SQ1	0.812
	SQ2	0.829
	SQ3	0.804
	SQ4	0.791
Perceived stress	PS1	0.824
	PS2	0.846
	PS3	0.833
	PS4	0.818
Recovery experience	RE1	0.861
	RE2	0.874
	RE3	0.846
	RE4	0.832
Flow experience	FL1	0.873
	FL2	0.882
	FL3	0.851
	FL4	0.836
Athlete burnout	AB1	0.882
	AB2	0.871
	AB3	0.856
	AB4	0.841

Results from the CFA reveal that the measurement model is a good fit to the observed data based on several indices. Specifically, the CFI and TLI (both > 0.957 and 0.948, respectively) surpass the benchmark criteria for an excellent incremental fit of the model compared to the null model. Furthermore, the RMSEA (0.049) suggests a very good approximation of the model to the population covariance structure, whereas the SRMR (0.041) attests to low residual correlation differences between the observed and estimated correlations.

### 4.4 Correlation analysis

**FIGURE 4:**
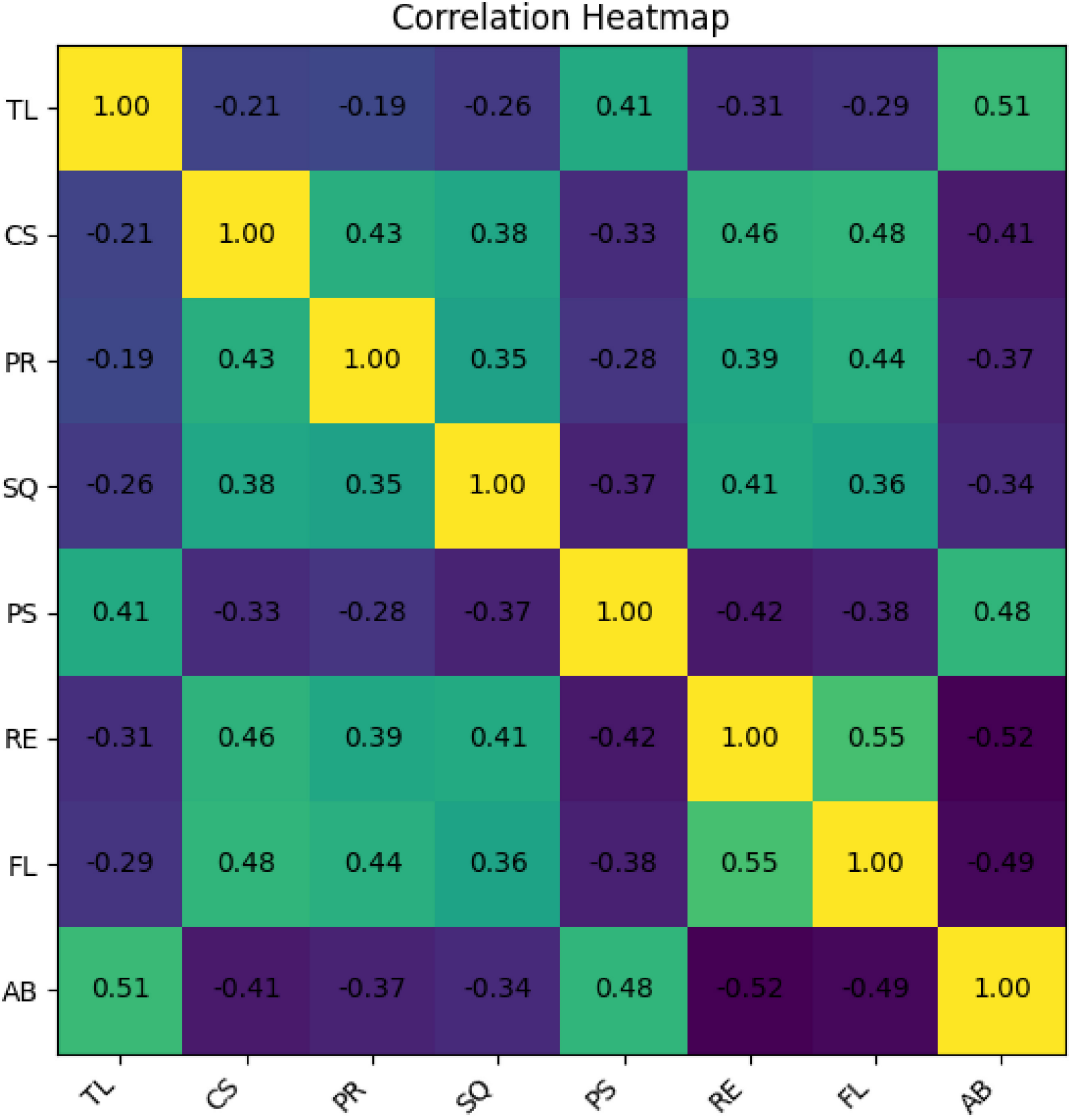
Correlation heatmap.

The correlation table in Table 4 shows the interrelations between the variables in the study. The findings show that there are quite a few strong relationships that are in line with the theoretical framework put forward. There was a moderate positive correlation between training load and athlete burnout (*r* = 0.51), indicating that the intensity of training is positively related to athlete burnout. On the same note, perceived stress had a positive relationship with burnout (*r* = 0.48), demonstrating that the greater the psychological pressure, the more it leads to burnout symptoms. On the other hand, the negative correlation between burnout and coaching support, resilience, sleep quality, recovery experience, and flow experience was observed. Recovery experience showed the most negative correlation with burnout (*r* = −0.52), then flow experience (*r* = −0.49) and lastly coaching support (*r* = −0.41). The findings indicate that positive environments and effective recovery systems are significant in eliminating burnout among the youth athletes (Figure 4).

**TABLE 4:** Correlations among study variables.

Variable	TL	CS	PR	SQ	PS	RE	FL	AB
Training load	1	1	1	1	1	1	1	1
Coaching support	−0.21							
Psychological resilience	−0.19	0.43						
Sleep quality	−0.26	0.38	0.35					
Perceived stress	0.41	−0.33	−0.28	−0.37				
Recovery experience	−0.31	0.46	0.39	0.41	−0.42			
Flow experience	−0.29	0.48	0.44	0.36	−0.38	0.55		
Athlete burnout	0.51	−0.41	−0.37	−0.34	0.48	−0.52	−0.49	

### 4.5 Hypothesis testing and structural model

The proposed hypotheses about the relationships between the variables were tested using structural equation modeling. The findings, as shown in Table 5, show that all the hypothesized direct relationships were statistically significant. The positive impact of training load on athlete burnout (β = 0.342, *p* < 0.001) supported H1. The effect of coaching support on burnout showed that H2 is true (β = −0.281, *p* < 0.001). Psychological resilience was also found to have a negative association with burnout (β = −0.196, *p* < 0.001), which supported H3, whereas sleep quality had a negative relationship with burnout (β = 0.173, *p* < 0.001), which supported H4. The perceived stress was strongly positively associated with burnout (β = 0.298, *p* < 0.001), which supports H5. Besides that, recovery experience (β = −0.324, *p* < 0.001) and flow experience (β = −0.276, *p* < 0.001) were both found to have significant negative impacts on athlete burnout, indicating the support of H6 and H7. The findings demonstrate that psychological recovery and the best performance experiences has play a critical role in alleviating burnout among youth athletes.

**TABLE 5:** Structural path coefficients.

s	Path	β	S.E	C.R	*P*-value	Result
H1	Training load → Burnout	0.342	0.052	6.58	<0.001	Supported
H2	Coaching support → Burnout	−0.281	0.048	−5.84	<0.001	Supported
H3	Resilience → Burnout	−0.196	0.043	−4.56	<0.001	Supported
H4	Sleep quality → Burnout	−0.173	0.041	−4.12	<0.001	Supported
H5	Perceived stress → Burnout	0.298	0.047	6.02	<0.001	Supported
H6	Recovery → Burnout	−0.324	0.049	−6.45	<0.001	Supported
H7	Flow → Burnout	−0.276	0.046	−5.96	<0.001	Supported

### 4.6 Mediation analysis

**FIGURE 5:**
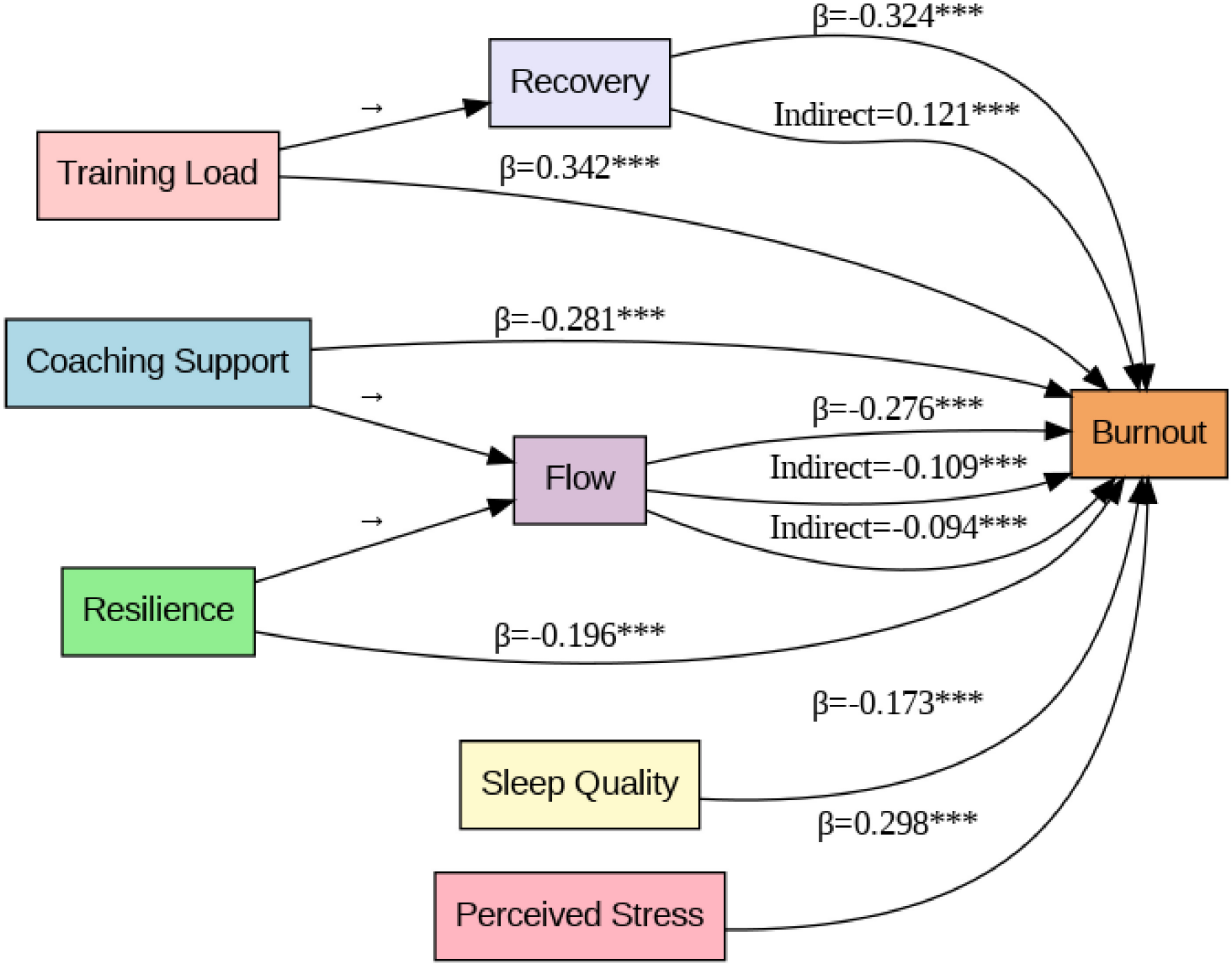
SEM pathway diagram. ***p < 0.001.

Bootstrapping was done to determine the mediating effects of recovery experience and flow experience. The findings in Table 6 show that recovery experience was a significant mediator of the correlation between training load and burnout, with an indirect effect of 0.121 (95% CI: 0.067183, *p* < 0.001), which supported H8. Likewise, flow experience mediated the connection between the coaching support and the burnout and the indirect effect of flow experience was −0.109 (95% CI −0.158 to −0.061, *p* = 0.001), which supported H9. Since the confidence intervals were not in the range of zero, the mediation effects were found to be statistically significant (Figure 5).

**TABLE 6:** Indirect effects.

Path	Indirect effect	Boot SE	95% CI	*P*-value
Training load → Recovery → burnout	0.121	0.029	0.067–0.183	<0.001
Coaching support → Flow → burnout	−0.109	0.026	−0.158 to −0.061	<0.001

### 4.7 model fit evaluation

The general structural model had a good fit to the observed data. According to Table 7, the ratio of chi-square to degrees of freedom (2/df) was 2.18, which is less than the recommended value of 3.0. Other model fit measures were also acceptable, such as CFI = 0.947, TLI = 0.939, RMSEA = 0.051 and SRMR = 0.047. These findings suggest that the suggested structural model is sufficient to show the relationships between the variables of the study.

**TABLE 7:** SEM model fit.

Fit index	Study value
χ^2^ / df	2.18
CFI	0.947
TLI	0.939
RMSEA	0.051
SRMR	0.047

### 4.8 Multilevel regression analysis

**FIGURE 6:**
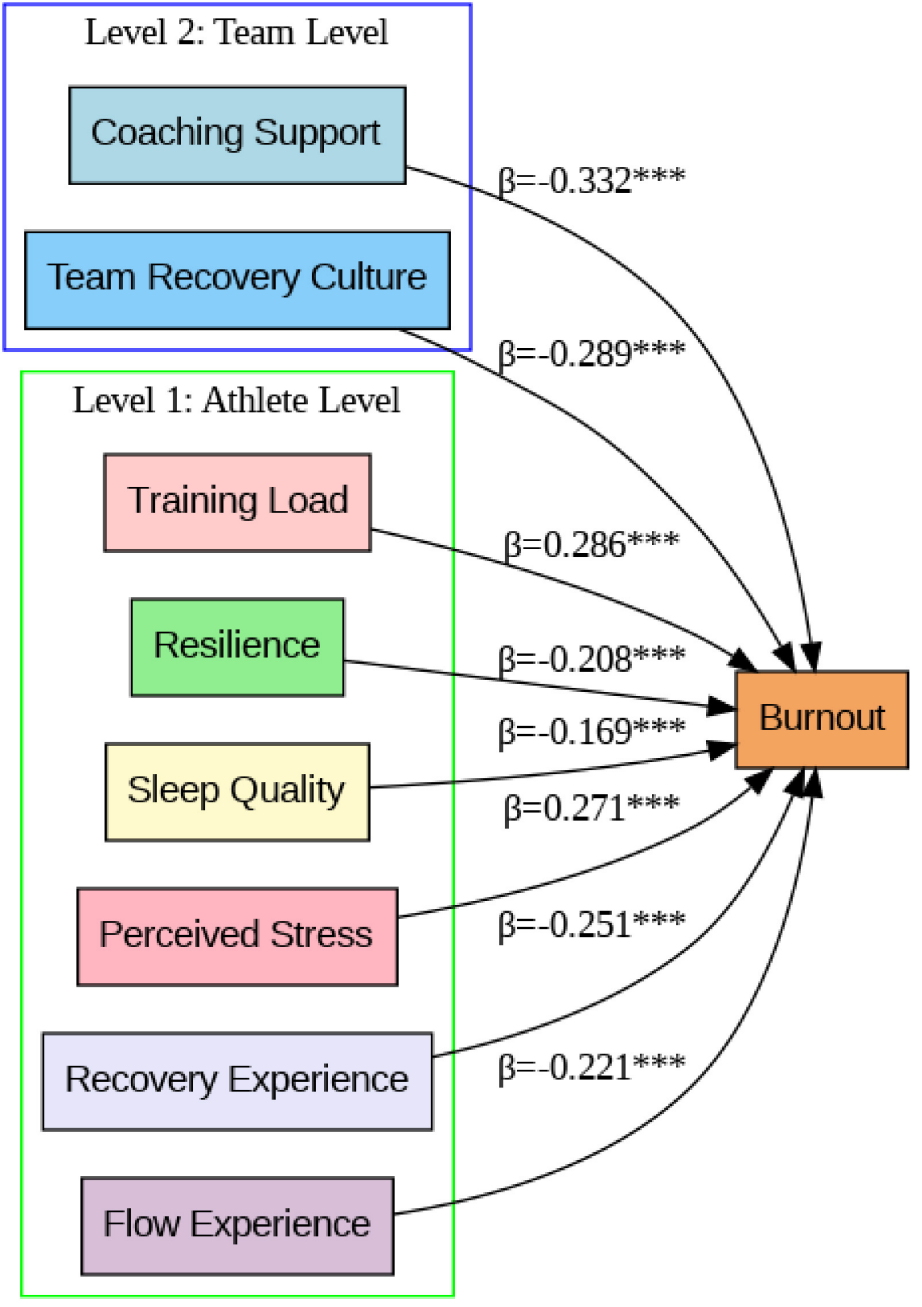
Multilevel pathway. ***p < 0.001.

The hierarchical nature of the data, with athletes being distributed in teams, was taken into consideration, and the multilevel regression analysis was performed. As determined in Table 8, there are multiple athlete-level variables that were significantly related to burnout. Training load (β = 0.314, *p* < 0.001) and perceived stress (β = 0.298, *p* < 0.001) had a positive effect on burnout, and resilience (β = −0.231, *p* < 0.001), sleep quality (β = −0.187, *p* < 0.001), recovery experience (β = −0.276, *p* < 0.001) and flow experience ( = −0.243, *p* < 0.001). At the team level, coaching support (β = −0.332, *p* = 0.001) and team recovery culture (β = −0.289, *p* = 0.001) had a significant impact to lower the level of burnout. The intraclass correlation coefficient (ICC = 0.249) demonstrates that about 24.9% of the variance in burnout was on the team level, which suggests the significance of the context of the team in determining the psychological outcome of athletes Table 9. The random slope model also confirmed that training load differed considerably among teams, which points to the fact that coaching settings and training systems have an impact on how athletes are exposed to workload-related stress Table 10. All these findings show that the individual psychological variables, as well as the environmental conditions of teams, are important factors in athlete burnout in youth high-performance athletes (Figure 6).

**TABLE 8:** Multilevel regression analysis predicting athlete burnout.

Predictor variables	Model 1: athlete-level model			Model 2: full multilevel model		
	β	SE	*P*-value	β	SE	*P*-value
Level 1: athlete-level predictors						
Training load	0.314	0.041	<0.001	0.286	0.039	<0.001
Psychological resilience	−0.231	0.037	<0.001	−0.208	0.036	<0.001
Sleep quality	−0.187	0.034	<0.001	−0.169	0.033	<0.001
Perceived stress	0.298	0.042	<0.001	0.271	0.040	<0.001
Recovery experience	−0.276	0.038	<0.001	−0.251	0.037	<0.001
Flow experience	−0.243	0.036	<0.001	−0.221	0.035	<0.001
Level 2: team-level predictors						
Coaching support	–	–	–	−0.332	0.061	< 0.001
Team recovery culture	–	–	–	−0.289	0.057	< 0.001
Model statistics						
Intercept	3.841	0.114	< 0.001	3.526	0.132	< 0.001
Athlete-level variance	0.642			0.571		
Team-level variance	0.213			0.149		
Intraclass correlation (ICC)	0.249			0.207		
−2 Log likelihood	1284.51			1236.28		
AIC	1298.63			1248.79		

**TABLE 9:** Random slope multilevel model results.

Fixed effects	Coefficient (γ)	SE	*T*-value	*P*-value
Intercept	3.512	0.129	27.21	<0.001
Training load	0.276	0.038	7.26	<0.001
Psychological resilience	−0.206	0.037	−5.57	<0.001
Sleep quality	−0.168	0.034	−4.89	<0.001
Perceived stress	0.269	0.042	6.41	<0.001
Coaching support (team level)	−0.318	0.058	−5.47	<0.001

**TABLE 10:** Random effects.

Random effect	Variance	SE	χ^2^	*P*-value
Intercept (team level)	0.154	0.041	18.37	<0.001
Training load slope	0.082	0.023	12.16	<0.01
Residual (athlete level)	0.548	0.029	–	–

### 4.9 Thematic analysis results

Qualitative procedures were employed through semi-structured interviews that involved 35 youth high-performance athletes and 12 coaches from elite training centers in Guangdong Province, China. The purpose of the interviews was to investigate psychological processes concerning burnout, recovery, flow, and training experiences. Interviews were conducted based on an interview protocol that was formulated on the basis of Self-Determination Theory, Flow Theory, Conservation of Resources Theory, and some major quantitative findings. All the interviews were carried out in Mandarin, recorded in audio format, transcribed verbatim, and translated using a back-translation approach. Reflexive thematic analysis based on Braun and Clarke’s six steps was employed for data analysis, facilitated by NVivo software. Open coding was conducted independently by two coders who generated codes through several readings of the transcriptions. Intercoder reliability was measured using Cohen’s Kappa, which yielded κ = 0.84, showing good intercoder reliability. Axial coding was conducted to cluster related codes into higher-level categories by comparing and discussing among the two coders; any discrepancies were resolved through consultations with a third expert researcher. Four major themes were identified inductively and deductively: psychological strain and training intensity, recovery and emotional regulation, flow and optimal performance, and coaching environment and teamwork.

#### 4.9.1 Theme 1: psychological strain and intensity of training

One theme that came out strongly after the interviews was the connection between rigorous training routines and psychological stress. Most sportspeople explained how hard elite sport training is, pointing out the physical and emotional stress involved in being able to uphold high performance levels. Long periods of training, repetitive interactions with the coaches and institutions were often cited by the participants as the causes of stress. Some of the athletes reported that the cumulative effect of these demands has at times led to fatigue, frustration and emotional exhaustion. One of the athletes described it as follows: “*During the competition season, we train twice a day, nearly every day. There are times I am physically exhausted, but the psychological stress is even more difficult since you understand the coach has been judging you to achieve the results*.” The other athlete also contemplated the psychological impact of the constant performance pressure: “*I am afraid that I have been forced out of the team in the event I underperform in training. This pressure has made training seem overwhelming*.” These reports show that training intensity is not a physical burden, but it is also a mental strain that affects the emotional conditions of athletes. Some athletes also mentioned that it is a challenge trying to strike a balance between training, academic work and social life, which only added to the stress. One of the participants referred to the following challenge: “*It is not easy to combine school and training. The combination of the two makes me mentally tired when both are demanding*.” In general, the results indicate that training demands and performance pressure are excessive factors that add to psychological stress, which has made people more susceptible to burnout.

#### 4.9.2 Theme 2: recovery practices and emotional regulation

The second theme indicated the significance of recovery experiences in ensuring psychological balance. Athletes often talked about various ways in which they had been restored to their usual level of functioning after training and competition. Such recovery strategies involved rest, relaxation, and mental disengagement with sport-related stress. The respondents insisted that proper recovery enabled them to replenish their energy, rebuild motivation, and remain emotionally stable. The importance of recovery in the management of training fatigue was described by one athlete who said, “*After a very intense training week, I attempt to relax by listening to music or friends. It makes me forget about training a bit and get back with a fresh mind*.” Another sports person has stressed the value of sleep in physical and psychological rest: “*Good sleep is huge. Sleeping properly makes me feel more concentrated and not stressed during training*.” Some of the athletes also stated that recovery experiences enabled them to be able to reflect on their performance without being too critical of themselves. One of the participants elaborated: “*In my free time between training, I has reason more clearly on what I need to work on rather than being frustrated*.” Coaches also appreciated the importance of rest intervals toward maintaining athlete fitness. One coach pointed out: “*When the athletes are not able to recover properly, their performance decreases, and the athlete is mentally fatigued. Training is important as recovery*.” These insights suggest that recovery practices are very important in restoring psychological resources and avoiding emotional depletion among youth athletes.

#### 4.9.3 Theme 3: flow experiences and optimal performance

The third theme that came out of the interviews was the experience of flow in the training and competition. Sportsmen often told of occasions when they experienced a total absorption in their performance, when their concentration was complete, they enjoyed what they were doing, and experienced control. Such experiences were usually related to top performance and good emotional conditions. One of the athletes had a typical flow experience when competing: “*I have had the impression that everything is going on spontaneously, and I am totally focused during a match. I do not consider errors or stress, and the game is just flowing*.” Another respondent added emotional pleasure that comes along with these experiences: “*I am excited and motivated when I am completely involved in training. It makes me understand why I am fond of this sport*.” Athletes also described flow experiences to have enabled them to defeat stress and remain motivated even in the face of rigorous training regimes. And, as one of the athletes explained: “*There are times when the training becomes challenging, and it all works. Those are the times that justify the struggle*.” These stories imply that flow experiences are highly effective psychological assets that make sport activities more enjoyable and engaging. Flow experiences has assist athletes to continue to take part in high-performance sport in the long term by raising positive emotions and intrinsic motivation.

#### 4.9.4 Theme 4: coaching environment and team support

The last theme was the modulation of coaching behavior and team environment as to its impact on the psychological functioning of athletes. The participants were consistent in highlighting that positive relationships with their coaches and positive team atmospheres helped them feel confident, motivated, and emotionally well. The athletes who reported that their coaches were supportive and empathetic said that they felt more comfortable when they raised their concerns and asked for advice. According to one athlete, the role of supportive coaching is important: “*When my coach provides positive feedback and motivation, I feel more confident and inspired to do better*.” One of the athletes emphasized the role that coaches play in helping to become emotionally resilient: “*Sometimes, training is difficult, but when the coach explains everything and helps us, it becomes easier to deal with the pressure. Relationships in the team were also declared as a vital support source*.” Athletes stated that the support of their teammates helped them to overcome the training problems and stay motivated. One of the participants said, “*In training with each other, we push each other. If one is exhausted or depressed, other members of the team have to ensure that he or she remains optimistic*.” Coaches also recognized the need to establish a supportive team culture that is supportive. One coach said, “*A good team atmosphere makes athletes feel safe and at ease. Once they have trust in the coaching staff and fellow teammates, they has perform better and have reduced stress*.” Altogether, this theme underlines the great importance of coaching practices and team dynamics in determining the psychological experiences of athletes. It seems that supportive coaching relationships and team work environments contribute to resilience, motivation, and emotional stability among youth high-performance athletes.

### 4.10 Integration of findings

The combination of quantitative and qualitative results gives a global insight into the psychological process that plays a role in burnout among youth high-performance athletes. The quantitative outcomes showed that training load and perceived stress had a strong impact on the athlete’s burnout, whereas coaching support, psychological resiliency, sleep quality, recovery experiences, and flow experiences had a strong impact on the level of burnout. Structural equation modeling further showed that recovery experience mediated the relationship between training load and burnout, and flow experience mediated the relationship between coaching support and burnout, and multilevel analysis showed that athlete- and team-level factors are both important contributors to the outcome of burnout. These statistical results underscore the issue of the complicated interaction between environmental requirements and psychological resources in determining athlete wellbeing.

The qualitative results are supportive and enriching to these statistical correlations as they show the ways athletes subjectively perceive the training pressures and the psychological coping mechanisms. The qualitative results that increased training load and stress have a positive relationship with burnout are directly supported by interview accounts under the theme of training intensity and psychological strain. The positive statistical correlation between training load and burnout is contextually clarified by the reports of fatigue, the stress of maintaining performance, and emotional burnout by athletes. On the same note, the topic of recovery practices and emotional regulation supports the mediating role of recovery experience that has been found in the quantitative analysis. Athletes named rest, relaxation, and social interaction as the key processes of energy restoration and psychological fatigue.

The flow experiences and optimal performance theme further indicates why there was a negative correlation between flow and burnout in the structural model. The flow experience has provided psychological engagement and resilience as evidenced by the descriptions of deep focus, pleasure, and enjoyment of the experience by athletes, which occur during the highest moments of their performance. Lastly, the coaching environment and team support theme is also consistent with the multilevel results, which indicate that coaching support has a significant negative impact on burnout. Athletes pointed out that positive team climates and supportive coaching behaviors are important to boost confidence, motivation and emotional stability.

Altogether, the synthesis of results reveals that burnout in youth high-performance athletes is determined by both the conditions of structural training and psychological experiences in the sport settings. Quantitative findings define the statistical connections between variables, whereas qualitative knowledge indicates the experience behind the connections. The mixed-methods approach together is able to offer a deeper and more comprehensive insight into the mental health of athletes in the elite sport systems.

## 5 Discussion

This study is a multilevel, mixed methods approach to investigate the relationships between training demands, psychological resources, recovery processes, psychological flow experiences, and athlete burnout in youth high-performance athletes in China. The results do not reflect a causal pathway but are viewed as psychological and contextual associations that exist within a nested sport environment. Overall, findings suggest that burnout is not only a result of high levels of training exposure but is better conceptualized as the result of psychological and relational resources in relation to perceived environmental demands. Higher perceived training load and stress were significantly related to higher burnout scores, while recovery experience, flow, coaching support, resilience and sleep quality were significantly negatively related to higher burnout scores. These patterns are similar to previous studies in sport psychology that have demonstrated that the demands of sport are likely to cause a burning out if they are perceived as chronically higher than the capacity of coping and recovery (Gustafsson et al., 2011; Raedeke and Smith, 2001). The present findings, however, do not only corroborate previous work, but also contribute to this literature by providing empirical evidence that the psychological recovery processes and optimal experience states could act as explanatory mechanisms between sport environments and burnout outcomes, in a context where intensity of training is structurally high, such as in an elite sport system of young athletes.

One of the main findings of this study is that recovery and flow experiences mediated the relationship between variables. One of the most important findings of this study was that variables were found to be mediated by the variables of recovery and flow experiences. The observed link between training load and burnout, which was in part mediated by recovery experience, highlights the consideration of recovery experience as stated in the Conservation of Resources Theory (Hobfoll et al., 2018) which regards the processes of replenishment of resources. Rather than as a passive byproduct of decreased workload, the findings argue that recovery takes place as an active psychological process that can help stop the build-up of stress and fatigue. Likewise, flow experience partially mediated the relationship between coaching support and burnout, suggesting that by creating a supportive coaching climate, flow experience could help prevent negative psychological states and promote positive psychological states that reverse the path toward disengagement and exhaustion. This builds on previous studies by Yiyi et al. (2025) and Zhang et al. (2024) that indicated that flow is not just a performance-enhancing state, but also a protective psychological pathway in larger motivational climates. It is also important to note that, since the data were cross-sectional, these mediation effects could be considered associational pathways.

These findings also highlight the importance of psychological factors like resilience, sleep quality, and perceived stress for athlete wellbeing. Resilience was consistently negatively related to burnout, as it is a protective psychological ability that enables athletes to respond to the constant training load. In line with the ideas presented by Mei et al. (2025), resilience can be conceptualized as a dynamic resource that engages with stressors and coping mechanisms in the environment. Likewise, the quality of sleep was a significant correlate of burnout, suggesting the importance of physiological recovery in psychological functioning. The present results suggest that sleep could be incorporated into the wider psychobiological frameworks that could affect emotional regulation and stress perception, whereas previous research has largely looked at sleep as a background recovery variable. The relationship between perceived stress and burnout, on the other hand, was positive, which is consistent with the stress appraisal models in sport psychology literature (Lazarus and Folkman, 1984), and indicated that stress effects might be exacerbated in high-performance fields for young people, where outside demands are high and last for extended durations (Guo et al., 2025).

The multilevel analysis also found that there was a significant amount of variance at the team level (24.9%), which suggests that contextual and organizational effects are important determinants of athlete psychological responses. The discovery cannot be read as a causal effect of coaching, but as an indication that the experience of athletes is shaped, to some extent, by common training environments. This aligns with the findings of Yang L. et al. (2024), who highlighted the importance of coaching climate and training organization for athlete’s wellbeing. The qualitative results brought depth of understanding in the interpretation of the quantitative results by providing a picture of the athletes’ experiences of coaching practices, training intensity and team climate as shared psychological environments that influence athletes’ stress and recovery patterns. The study had not been formally tested for differences between the demographic variables (gender, age, sport modality), however, and thus does not necessarily show whether these associations differ within these subgroups.

Combining the qualitative and quantitative results added depth to the interpretation by providing a visualization of the statistical associations in real-life situations. Athlete responses of fatigue and emotional stress appeared to support the positive relationship between training load and burnout, whereas reports of rest, detachment, and emotional regulation seemed to support the protective nature of recovery experience. Analogously, stories of immersive engagement and fun at optimal performance confirmed the negative link between flow and burnout, as did stories of coach actions and team climate for relationship support. As a whole, these results indicate that the development of burnout in youth elite sport is a result of the continuous negotiation of the environment and psychological meaning-making processes, and not from any single factor.

### 5.1 Implications

This study has a number of theoretical and practical implications. Theoretically, the research adds to the literature of sport psychology by bringing together the burnout, recovery, and flow in a multilevel framework. Whereas some past studies have looked at these constructs individually, the current study establishes the interaction of these constructs to affect the mental health of the athletes. Recovery and flow were identified as mediators, which makes a new understanding of how the training settings influence the psychological outcomes to expand the previous models of burnout and motivation.

In practical terms, the results indicate that there is a need to focus on balanced training programs that are focused not only on performance but also on the wellbeing of the athletes. To avoid burnout, coaches and sport organizations are to adopt systematic recovery interventions such as sufficient rest, mental support and sleep control. Also, by encouraging the conditions that stimulate flow experience (e.g., autonomy-supportive coaching and developing interesting training conditions), performance and wellbeing have been improved. Coach education programs should also focus on interpersonal skills, communication and athlete-centered training methods, which is implied by the significance of coaching support.

### 5.2 Limitations and future research

However, there are also some limitations associated with the current investigation. Firstly, the application of self-report tools can generate response and social desirability biases, thus creating an upward bias in the relationships between constructs. Secondly, due to the cross-sectional nature of the study, no conclusions about causality can be made; therefore, all the relationships found must be treated as associations. Thirdly, the sample of athletes in the study was recruited in Guangdong Province; hence, the results cannot be generalized to other regions, especially considering particularities of the Chinese elite sport system. Lastly, some contextual factors, such as gender, type of sport, and athletes’ experience, may have impacted the relationships found in the present study.

## 6 Conclusion

This paper presented an in-depth analysis of burnout, recovery, and flow in youth high-performance athletes in a multilevel mixed-methodology study. The results showed that the individual psychological factors, as well as the environmental conditions of a team, play an essential role in the occurrence of athlete burnout. In particular, the training load and perceived stress were characterized as the risk factors, whereas recovery experiences, flow states, resilience, sleep quality, and coaching support were also proposed as the key protective means. The research also found that recovery and flow are significant mediating variables, which show the role of psychological involvement of athletes and their experiences of rest in determining their reaction to training needs. The multilevel analysis highlighted the importance of team context, that a favorable coaching climate and a healthy team culture are critical in preventing burnout and enhancing wellbeing. These findings were enhanced by qualitative insights that demonstrated lived experiences of athletes regarding stress, recovery and optimal performance. On the whole, this work has been considered as a contribution to the increased range of literature in the field of sport psychology since it combines several psychological concepts into a single model of athlete wellbeing. It emphasizes the necessity of holistic and athlete-focused training models, which consider both the performance requirements and mental health factors, as the eventual outcome of creating sustainable development in high-performance youth sport systems.

## Data Availability

The original contributions presented in this study are included in this article/supplementary material, further inquiries can be directed to the corresponding author.
